# Geometry aware neural radiance fields for freehand ultrasound reconstruction

**DOI:** 10.1088/2057-1976/ae74d7

**Published:** 2026-07-01

**Authors:** Yimeng Dou, Yin Li, Tomy Varghese

**Affiliations:** 1 Department of Electrical and Computer Engineering, UW-Madison, Madison, WI 53706, United States of America; 2 Department of Medical Physics, University of Wisconsin (UW) school of Medicine and Public Health, Madison, WI 53705, United States of America; 3 Department of Biostatistics and Medical Informatics, University of Wisconsin (UW) school of Medicine and Public Health, Madison, WI 53705, United States of America; 4 Department of Computer Sciences, UW-Madison, Madison, WI 53706, United States of America

**Keywords:** freehand ultrasound reconstruction, implicit neural representation, neural rendering

## Abstract

Reconstructing 3D volumes from 2D freehand ultrasound (US) is a challenging task. During reconstruction, the ensuing overlap between sweeps can cause multiple pixels to be assigned to the same voxel, so the accurate alignment of these sweeps is critical. In recent years, implicit representation methods, such as neural radiance fields (NeRFs), have been utilized for modeling the 3D scene as a continuous volumetric function learned from 2D B-mode images. However, NeRF-based methods are also highly sensitive to errors in camera or transducer poses, a challenge that is particularly pronounced in freehand US with misregistration between overlapping sweeps, leading to severe reconstruction artifacts. To address this challenge, we propose geometric aware US NeRF (GAU-NeRF) by introducing a gradient reweighting strategy to reduce gradient fluctuations from noisy poses during early training iterations and to stabilize the optimization process. GAU-NeRF results in improved accurate pose refinement and improved reconstruction quality. Our method significantly outperforms existing baseline models on both simulated and *in vivo* US datasets, achieving substantial gains across multiple metrics, including up to 132% increase in the peak signal-to-noise ratio, 133% improvement in the structural similarity index measure, and 350% reduction in the learned perceptual image patch similarity.

## 1. Introduction

Three-dimensional (3D) ultrasound (US) reconstruction aims to create a detailed and comprehensive 3D representation of the scanned internal structures, utilizing a series of two-dimensional (2D) US B-mode (Brightness mode) images collected by sonographers [1]. This is essential to obtain an anatomical representation for treatment planning and for navigation and orientation of biopsy or ablation devices, when precise targeting of tumors or vasculature is critical [2, 3]. Acquisition methods using “wobbler” or “matrix array” transducers for 3D US reconstruction often suffer from a small field of view, low volume frame rates during acquisition, or shallow depth of the imaged regions [4, 5]. In addition, interpolation errors can affect any US compounding method, due to limited imaging planes [6].

Freehand 3D US combines 2D US scanning with 3D motion tracking to enable direct *in vivo* measurements of tissue structures. It creates a stack of 2D B-mode images by capturing sequential US scans while simultaneously tracking the transducer’s position and orientation (also referred to as the transducer pose) using position sensors. It offers significant benefits due to its ease of use during image acquisition and reconstruction [7]. For larger target regions, the use of a freehand system is essential, requiring several sweeps with the transducer to cover the entire region of interest to capture the underlying 3D structure without gaps [8–10]. However, multisweep freehand acquisition creates additional reconstruction challenges. Tissue deformation during scanning, variable acoustic properties, patient respiration, and artifacts introduced by transducer, represent some of these challenges [1, 11, 12] hinder the ability to establish consistent spatial correspondences and maintain robust tracking during reconstruction.

**Figure 1.**
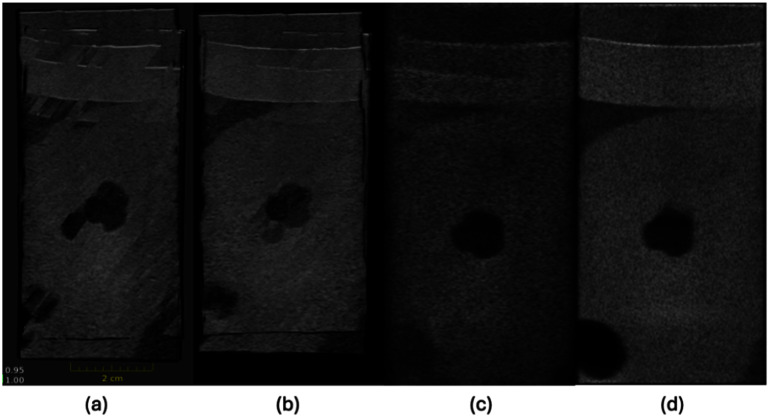
(a) Overlap of two freehand US images acquired during two different sweep sequences for a tissue-mimicking abdominal phantom, where misregistration between two sweeps occurs. (b) Reconstruction using distance weighting [4], which shows incorrect 3D geometry. (c) Reconstruction result from Ultra-NeRF. (d) Reconstruction with our GAU-NeRF method, which correctly recovers the underlying 3D structure.

Misalignment in US sweeps, depends on the accuracy of tracked position information, which often results in inconsistent intensities for the same spatial location [8, 13]. Merging these sweeps into a single 3D volume creates artifacts that resemble motion blur artifacts in 2D photography [8]. As shown in figure 1(a), inaccurate overlap in the registration of 2D US images from multiple sweeps can occur. Figure 1(b) illustrates a reconstruction example using distance-weighting (DW), a voxel-based reconstruction method [4] when inaccurate transducer poses are presented.

An alternative approach is to estimate transducer poses directly from a sequence of 2D B-mode images. These image-based methods [1, 14–16] eliminate the need for external tracking hardware, making them compatible with all US imaging systems. Moreover, recent studies have demonstrated promising performance, positioning these techniques as practical, increasingly viable solutions for sensorless freehand 3D US reconstruction. However, for image-based methods, small prediction errors in transducer poses can accumulate, leading to significant drift over time and ultimately causing major misalignment of 2D US images to the 3D space [17]. Several learning-based methods have recently been proposed to reconstruct 3D volumes from misaligned transducer poses [18, 19]. These methods require a large amount of annotated data between the 2D images with a predefined 3D atlas, which limits their usage.

Recent advancements in neural radiance fields (NeRFs) offer an appealing solution for freehand 3D US reconstruction. NeRF represents a 3D scene as a set of continuous, differentiable signals parameterized by a neural network, e.g. multi-layer perceptrons (MLPs), enabling implicit recovery of the 3D scene [20]. Using MLPs to replace the traditional grid-based 3D representations for US reconstruction has been previously discussed in [21–24], and Ultra-NeRF [25] was developed for US reconstruction from overlapping sweeps. The quality of Ultra-NeRF reconstruction is based on accurate transducer poses without any mis-registration errors associated with the 2D US images and without any artifacts [26, 27], resulting in degraded results with real data (see figure 1(c)). Previous work has attempted to address this issue for 3D reconstruction with natural RGB images [28–31] using gradient-based optimization. Methods like bundle adjusting NeRF (BARF) directly optimize the global geometric transformation of camera poses. However, such approaches can produce suboptimal solutions and incorrect reconstruction results [30, 32, 33]. More importantly, none of these approaches have not been adapted for US reconstruction.

Therefore, there is a fundamental gap in current approaches: existing NeRF-based methods designed for natural imaging do not account for US specific imaging physics and constraints. Unlike the traditional camera, US transducers emit directional acoustic waves that propagate through tissue with depth dependent attenuation, reflection, and scattering. Additionally, anatomical constraints impose strict limitations on valid transducer positions and orientations. Sonographers or physicians cannot arbitrarily position the transducer as one would with a camera, as the human subject anatomy and imaging constraints have to be considered (details are described in chapter 3.3).

To bridge this gap, we propose a novel NeRF-based method: geometry aware US NeRF (GAU-NeRF), which can perform freehand 3D US reconstruction with multiple sweeps by jointly reconstructing the neural fields and registering transducer parameters. Inspired by Geometric Polyak–Ruppert averaging [34], a key technical innovation of GAU-NeRF is to reweight and smooth first-order gradients in earlier training iterations. This strategy seeks to reduce the variance during gradient updates, thereby limiting the negative impact of large gradient fluctuations while recovering neural fields from misaligned transducer poses. As shown in figure 1(d), our proposed model correctly recovers the underlying 3D structure while DW and Ultra-NeRF generate incorrect results. We demonstrate the effectiveness of our model using a simulated phantom [25], tissue-mimicking (TM) abdominal phantom, and *in vivo* knee datasets [24]. Our results demonstrate that GAU-NeRF significantly improves reconstruction quality by accurately recovering pose information than baseline methods. Meanwhile, reconstruction metrics, including the peak signal-to-noise ratio (PSNR), the structural similarity index measure (SSIM) and the learned perceptual image patch similarity (LPIPS) are improved by up to 132%, 133% and 350%, respectively.

## 2. Related work

### 2.1. Neural implicit representation for US

Implicit neural representations (INRs) have shown some success in US reconstruction and novel view synthesis. Yeung *et al* [19] used a deep neural network to learn mappings from 2D fetal brain images and estimated 3D positions in 3D space. Eid *et al* [18] addressed the issue of noisy position information in freehand US reconstruction. However, this method relied on [17] to predict the location of 2D US images in a 3D atlas with a spatial coordinate system, which is not generalizable and requires large amount of training data. Several other methods have been proposed to learn surface and shape information from US volumes [35–37]. Gu *et al* [21] showed that INR can be used for US view synthesis to create a new image. Song *et al* [23] demonstrated the feasibility of reconstructing carotid vasculature, while the spine was reconstructed by Li *et al* [22]. However, these types of INR algorithms require accurate transducer poses.

NeRF is a type of INR that performs novel view synthesis, a process of creating a new image of a 3D scene from a previously unseen camera/transducer pose. However, the vanilla NeRF was designed without considering US image formation model, leading to unrealistic US image rendering. Wysocki *et al* [25] further updated the physical rendering model to better reflect underlying US physics, referred to as Ultra-NeRF. Dgli *et al* [24] then developed Ultra-NeRF and incorporated 3D priors learned from a diffusion model into NeRF training, aiming to reduce reconstruction artifacts. UlRe-NeRF [38] was then proposed to improve the fidelity of reconstructions by modeling the behavior of US waves propagation in the medium. Gaits *et al* [39] studied the impact of encoding in terms of reconstruction realism. Critically, these methods assume that accurate transducer poses are provided as input. Unlike other INR methods, GAU-NeRF jointly recovers the underlying 3D structure from imperfect transducer poses from multiple sweeps.

### 2.2. Pose optimization

An alternative approach to external tracking hardware is to estimate transducer poses directly from a sequence of 2D B-mode images. Regression based methods for pose optimization train an encoder to estimate pose information from 2D images or 3D features. Methods like [1, 7, 14, 15, 40, 41] are designed to predict relative pose information with 2D US images by leveraging over–parameterized distributed representations of motions. Other learning based methods include pose optimization based on generative [32, 42, 43] and foundational [44] models. However, these methods require a large amount of tracked data, which is labor-intensive to curate.

Several image-based methods perform pose refinement directly. ImplicitCell [45] utilizes resolution cell modeling of US to improve the accuracy of poses. However, it assumes fully developed speckle, a condition that may not exist in the acquired images [7]. Esposito *et al* [46] proposed an approach to reduce noise and jitter from the electromagnetic tracking system, but the reconstruction result using this method depends on user-defined hyperparameters and is not designed for multiple freehand sweeps. Therefore, image-based tracking methods are complementary to, but insufficient for, stable freehand 3D reconstruction.

On the other hand, several methods have been proposed to jointly optimize camera/transducer pose and neural fields. BARF [31] was designed to bridge the gap by jointly optimizing NeRF and camera pose with a coarse-to-fine approach, starting with a rough scene and refining it progressively for better accuracy. Gaussian Activated NeRFs [47] utilizes different activation functions for pose optimization. SCNeRF [48] minimizes the ray-interaction reprojection error to optimize the camera’s extrinsic and intrinsic properties. Incremental CONfidence (ICON) [33] introduces an adaptive confidence measurement for optimizing poses without good initialization. L2G-NeRF [49] is designed for pose recognition by using a warp neural field to transform the learned query coordinate into global alignments. Those methods will likely struggle with US data, as they are dominated by speckle noise and highly dependent on view and transducer orientation.

### 2.3. The missing piece

To the authors’ best knowledge, no existing method explicitly addresses the instability in optimization when multiple US sweeps with imperfect poses overlap anatomical regions. This motivates our GAU-NeRF approach, which introduces a gradient reweighting strategy to reduce gradient fluctuations from contradictory multi-sweep observations during early training iterations, stabilizing joint optimization and enabling convergence to geometrically correct reconstructions despite multi-sweep misalignment.

## 3. Method

In this section, we describe our proposed GAU-NeRF for freehand 3D US reconstruction using multiple freehand sweeps. GAU-NeRF utilizes as input a sequence of 2D US images of a 3D structure acquired from multiple sweeps, with misregistration errors present between freehand sweeps. The goal for GAU-NeRF is to accurately learn the underlying 3D scene being reconstructed. We begin with an overview of the NeRF model and its variant for US (Ultra-NeRF). We then present our method to jointly optimize NeRF and transducer poses, describe its training loss and learning procedure, and provide implementation details.

### 3.1. Preliminaries: NeRF and Ultra-NeRF

**NeRF** [20] builds on a neural network that implicitly encodes volumetric information. This is done by mapping viewing angle and sampling location into color values $\hat{\textbf{c}}$ and volumetric density $\sigma$ for each 3D spatial location, allowing the network to output different representations for the same 3D point from different viewing angles. To train NeRFs, we sample pixels from the original images without ground truth (GT) density and color using ray marching. Each input pixel has the ray $\textbf{r}(h) = o+h\textbf{d}$ sampled at a timestamp $h$ where $o$ represents the ray’s origin and $\textbf{d}$ denotes the viewing direction. NeRF employs numerical integration based on the Gaussian quadrature rule to map these back to a novel image $\hat{I}$ and along the way of $\textbf{r}(h)$:

\begin{equation*} \hat{I} = \sum_i^N K_i \left(1-\exp\left(-\sigma_i\delta_i\right)\hat{c_i}\right),\end{equation*}

where

\begin{equation*} K_i = \exp\left(-\sum_{j = 1}^{i-1} \sigma_i\delta_i\right).\end{equation*}

$K_i$ is the accumulated ray transmittance along $r(h)$ partitioned into non-overlapping intervals $[h_i,h_{i+1}]$ and $\delta_i = h_{i+1}-h_i$. $N$ is the total number of intervals the ray is divided into. Equation (1) renders the neural field into a 2D image $\hat{I}$. NeRF is optimized using a photometric loss term between the GT image $I$ and $\hat{I}$.

**Ultra-NeRF** [25] was adapted from the original NeRF framework by utilizing a ray-tracing-based rendering formulation described in [50]. Unlike NeRF, Ultra-NeRF reconstructs a 3D representation by learning the view-dependent appearance and geometry directly from multiple 2D US sweeps and their transducer positions in a single training stage [25]. Using the same notation as before, each ray $\textbf{r}$ is associated with a single scan line, originating from $o$ located at the top of the image plane and extending in the direction $\textbf{d}$ at a distance $h$ away from the transducer. Along the ray, Ultra-NeRF samples several query points where one is represented as a 3D point $\hat{x}_j = \textbf{r}(h_j )$ in world coordinates at distance $h_j$ from $o$. Color values as well as volumetric density from NeRF are replaced with learnable US physics parameters: attenuation $\boldsymbol{\alpha}$, reflectance $\boldsymbol{\beta}$, boundary probability $\mathbf{p_{b}}$, scattering density $\mathbf{p}_{\mathbf{s}}$, and scattering intensity $\boldsymbol{\phi}$. The rendered US image $(I_i ) [\textbf{r}(h)]$ is defined as the sum of reflected $\hat{F}$ and backscattered $\hat{B}$ energy as $(I_i )[\textbf{r}(h)] = \hat{F_i } [\textbf{r}(h)]+\hat{B_i } [\textbf{r}(h)]$, with the assumption of an initial unit intensity $\hat{E_0}$ at the origin,

\begin{align*} \hat{F_i } &amp;= | \hat{E_0} \prod_{j = 0}^{h_{i-1}} \left[(1-\boldsymbol{\beta}(\textbf{r}(j))\cdot G(\textbf{r}(j)) \cdot \exp (- \int_{h_j}^{h_{i-1}} \boldsymbol{\alpha} \mathrm{d}t) \right]\nonumber \\ &amp;\quad \cdot \beta(\textbf{r}(h_{i-1})) | \cdot PSF (\textbf{r}(h_{i-1})) * S (\textbf{r}(h_{i-1})),\end{align*}

and

\begin{align*} \hat{B_i } &amp;= | \hat{E_0} \prod_{j = 0}^{h_{i-1}} \left[(1-\boldsymbol{\beta}(\textbf{r}(j))\cdot G(\textbf{r}(j)) \cdot \exp (- \int_{h_j}^{h_{i-1}} \boldsymbol{\alpha} \mathrm{d}t) \right] \nonumber \\ &amp;\quad\cdot \beta(\textbf{r}(h_{i-1})) | \cdot PSF (\textbf{r}(h_{i-1})) * (S (\textbf{r}(h_{i-1})) \cdot \boldsymbol{\phi} (\textbf{r}(h_{i-1}))).\end{align*}

Here, $PSF(\textbf{r})$ is a predefined 2D point-spread function. $G(\textbf{r})$ represents a boundary mask and $S(\textbf{r})$ is the scattering mask, parameterized by $\mathbf{p}_{\mathbf{b}}$ and $\mathbf{p}_\mathbf{s}$, respectively. The network $f$ can be summarized as $\textbf{y} = \left[\boldsymbol{\alpha},\boldsymbol{\beta},\mathbf{p}_{\mathbf{b}},\mathbf{p}_{\mathbf{s}}, \boldsymbol{\phi}\right] = \ f\left(\textbf{x};\boldsymbol{\Theta}\right)$ where $\boldsymbol{\Theta}$ is the network parameters and $\textbf{x}$ denotes the query point vector. Similar to NeRF, a 2D image can be generated by mapping the parameters with the position of transducer $\mathbf{P_i}$. For readers who are not familiar with this literature, we provide a diagram that illustrates the core components of US tissue interaction in appendix A.1.

### 3.2. Neural field representation for US

To represent 3D transducer poses, we adopted the parameterization described by Wang *et al* [28] by introducing rotation $\textbf{R}$ in a 3D rotation group $SO\left(3\right)$ and translation $\textbf{t}$ of the transducer location in the world coordinate system as

\begin{align*} \textbf{t} &amp;= \left[t_{x}, t_y, t_z\right]\in{\mathbb{R}}^3, \textbf{R} = \left[\mathbf{u}_{1}^{^{\prime}}; \mathbf{u}_{2}^{^{\prime}}; \mathbf{u}_{3}^{^{\prime}} \right],\nonumber\\ \mathbf{u}_{1}^{^{\prime}} &amp;= \left[1,-\theta_z, \theta_y \right], \mathbf{u}_{2}^{^{\prime}} = \left[\theta_z,1,-\theta_x\right],\ \mathbf{u}_{3}^{^{\prime}} = \left[-\theta_y, \theta_x, 1 \right].\end{align*}

Following the approach proposed by Zhou *et al* [51] and Agostinho *et al* [52], Gram-Schmidt orthogonalization strategy was adopt to transform the $\textbf{R}$ as follows:

\begin{equation*} \mathbf{u}_{1} = \ \frac{\mathbf{u}_{1}^{^{\prime}}}{\left|\mathbf{u}_{1}^{^{\prime}}\ \right|^2},\ \mathbf{u}_{2} = \frac{\left(\mathbb{I}-\mathbf{u}_{1}{\mathbf{u}_{1}}^\top\right)\mathbf{u}_{2}^{^{\prime}}}{\left|\left(\mathbb{I}-\mathbf{u}_{1}\mathbf{u}_{1}^\top\right)\mathbf{u}_{2}^{^{\prime}}\right|^2},\ \ \mathbf{u}_{3} = \ \mathbf{u}_{1}\ \times \mathbf{u}_{2}.\end{equation*}

**Figure 2.**
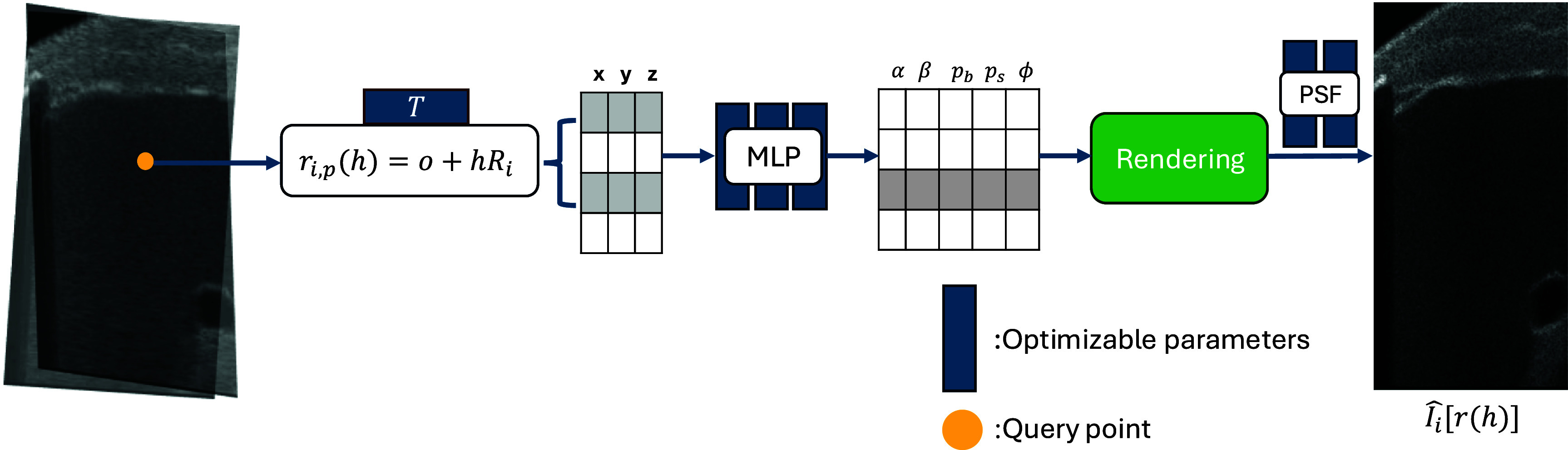
Schematic diagram of our proposed model. The ultrasound physics model dictates the ray origin, starting at the origin $o$, where the transducer contacts the skin surface. MLP estimates the parameters from a query point defined by their spatial location $t_i+h\mathbf{R_i}$ for $\mathbf{P_i}$. From those parameters, the pixel value at the query point can be generated as $\hat{I}\left[\mathbf{r_i}\left(h\right)\right]$. To train this model, photometric loss is used to measure the difference between the generated and ground truth image. Point spread function (PSF) as well as the pose parameters are optimized to improve the reconstruction quality.

$\mathbb{I}$ is the identity matrix. Then, each transducer pose can be expressed as a transducer-to-world transformation matrix, denoted as $\textbf{T} = \left[\textbf{R}\middle| \textbf{t}\right]$ belonging to the special Euclidean group $SE\left(3\right)$. Given the transformation matrix of the transducer, we can now formulate the rendering operation of NeRF for US as shown in figure 2. With the number of sweeps $W$ and the ray compositing function $g$, the grayscale value $\hat{I}$ of a 2D US image can be rewritten as $\hat{I} = g\left(\mathbf{y_1},\ldots,\ \mathbf{y_W}\right)$. Under a transformation matrix $\mathbf{T_i}$ associated with the transducer pose $\mathbf{P_i}$, the corresponding 3D query point $\mathbf{x_j}$ along the ray at depth $h_j$ is $x_j = \mathbf{r_i}\left(h_j\right) = \ \mathbf{t_i}+h_j\mathbf{R_i}$ with the current estimate of the transducer parameters. The network $f$ is evaluated at the sampled 3D point to obtain the corresponding learnable parameters $\left[\boldsymbol{\alpha},\boldsymbol{\beta},\mathbf{p}_{\mathbf{b}},\mathbf{p}_{\mathbf{s}},\boldsymbol{\phi}\right]$ that encode US physical signals. In the final phase, the synthesized greyscale value during the rendering phase can be described as the following equation

\begin{equation*} \hat{I}\left[\textbf{r}\left(h\right)\right] = g\left(f\left(\mathbf{t_1}+h\mathbf{R_1};\boldsymbol{\Theta}\right),\ldots,\ f\left(\mathbf{t_W}+h\mathbf{R_W};\boldsymbol{\Theta}\right)\right),\end{equation*}

from all sweeps that cover the pixel $p$.

### 3.3. Pitfalls in joint optimization of transducer poses with 2D US images

Given a collection of US sweeps, we aim to jointly find neural field parameters $\boldsymbol{\Theta}$ and the transducer pose $\textbf{T}$ (geometric transformation matrices) that minimize the photometric error $L_{photo}$ using mean square error (MSE) loss. Gradient-based optimization seeks to solve the problem with the objective:

\begin{equation*} \boldsymbol{\Theta}^\ast,\textbf{T}^\ast = \arg{\min_{\boldsymbol{\Theta},\textbf{T}}P{\left(\hat{I}\middle| I,\textbf{T}\right)}},\end{equation*}

where $\boldsymbol{\Theta},\textbf{T}$ denote model parameters and trainable camera parameters, $\hat{I}$ and $I$ denote reconstructed gray scale and its GT. According to the backpropagation process, equation (8) can be derived as:

\begin{equation*} \Delta = \sum_{i = 1}^{N}{\frac{\delta\widehat{I_i}}{\delta \mathbf{y_i}}\frac{\delta \mathbf{y_i}\left[\mathbf{r_i}\left(h\right)\right]}{\delta \mathbf{r_i}\left(h\right)}\frac{\delta \mathbf{r_i}\left(h\right)}{\delta \textbf{T}}}.\end{equation*}

The Jacobian of the network $\frac{\delta \mathbf{y_i}\left[\mathbf{r_i}\left(h\right)\right]}{\delta \mathbf{r_i}\left(h\right)}$ linearly relates to the change of parameters $\textbf{y}$ with $\mathbf{r_i}\left(h\right)$. Therefore, gradient-based optimization points out pose transformation matrix $\mathbf{T_i}$ can be updated for each iteration as:

\begin{equation*} \textbf{t}\ = \ \textbf{t}+\widehat{\left\{\Delta \textbf{t}\right\}},\ \textbf{R} = \widehat{\left\{{\Delta \textbf{R}}\right\}}\ \oplus \textbf{R},\end{equation*}

where $\oplus$ denotes an update on $SO\left(3\right)$.

**Figure 3.**
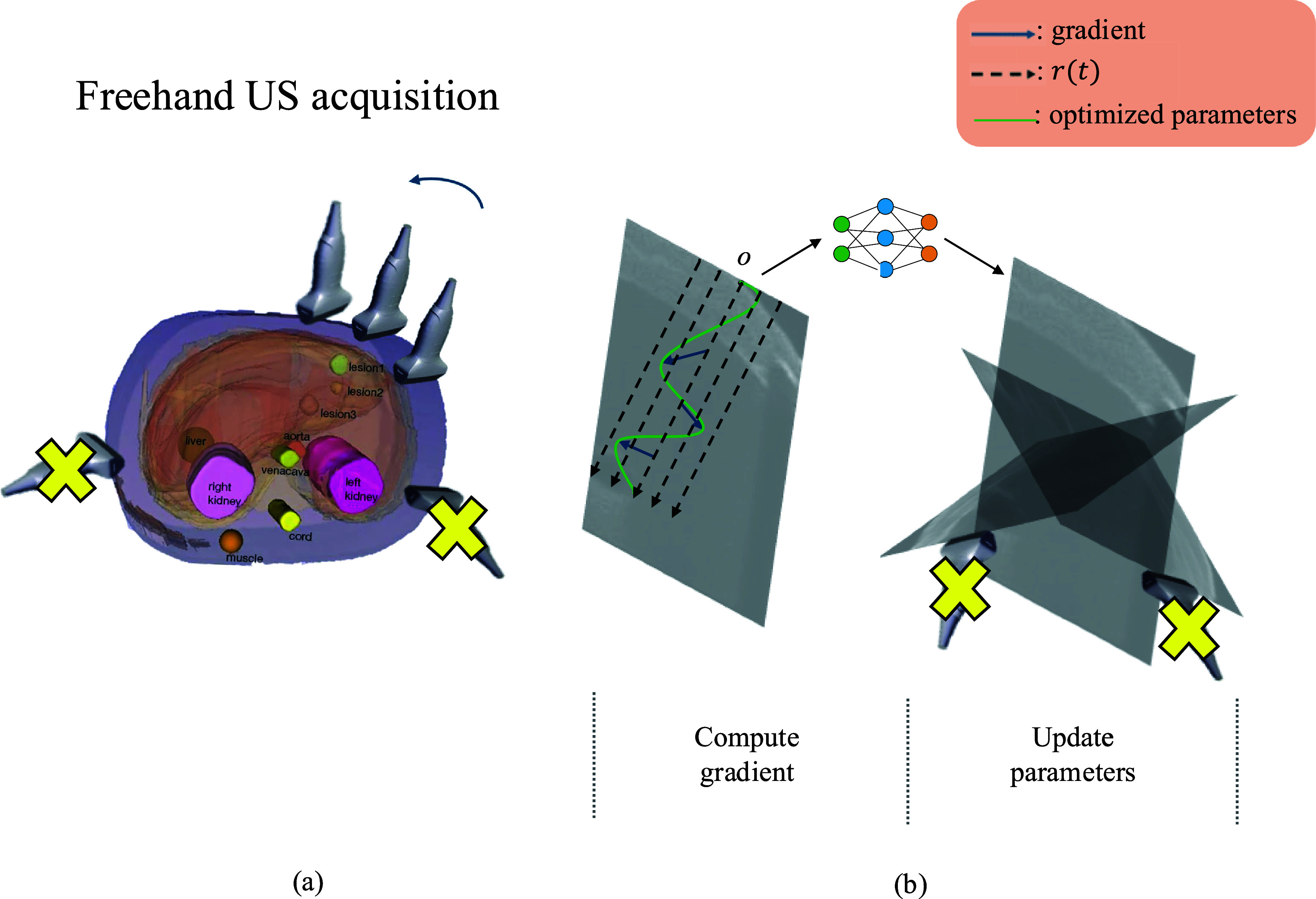
For freehand US acquisition, the possible transducer positions for a given scene are limited, as shown in (a). Following Ultra-NeRF, the transducer origin starts at the top of the image plane, with direction $d$ pointing along the scanline. Query weights from the MLP are obtained for scene properties along that ray at query points defined by their spatial location. (b) shows the problem with jointly optimized transducer position information. With limited constraints on possible transducer positions, the optimization could contribute to inaccurate pose estimations.

As the optimization is non-linear and non-convex, and contains several local minima, a single transducer pose update is vulnerable to local minima during the optimization process [53], resulting in potentially misleading learning signals. In US imaging, each 2D image is a cross-section of the body where the US transducer is placed, and several limitations restrict the possible position of the transducer, such as attenuation of the sound wave propagation in tissue, inaccessibility of the transducer due to physiological or anatomical constraints, specular reflection at oblique angles, or low image quality at large incidence angle with the skin surface. Figure 3 illustrates some of these limitations.

**Figure 4.**
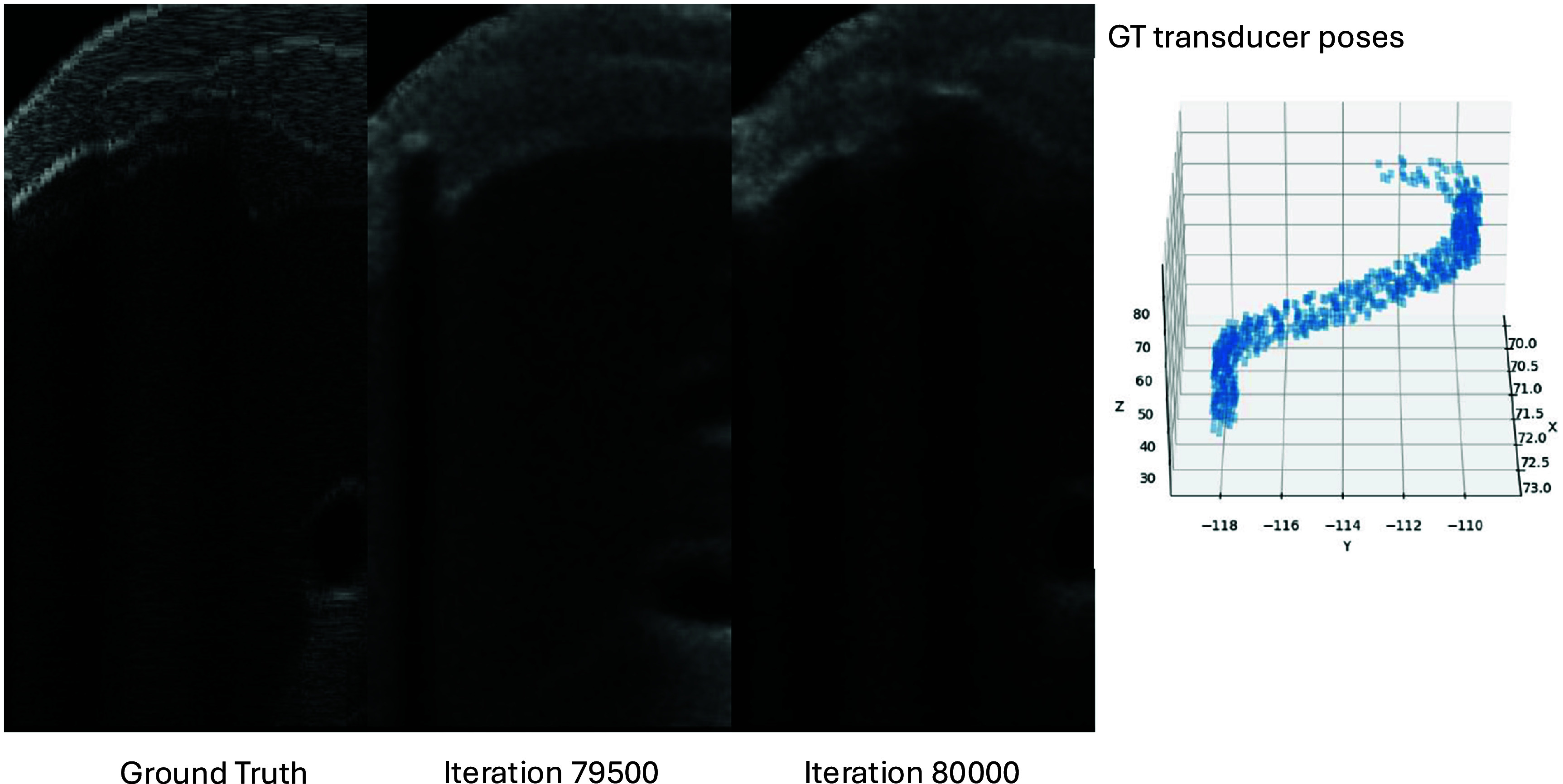
In order to acquire a large volumetric image with freehand US, the operator sweeps the transducer linearly over the scanning region. The sequence of poses from all sweeps is shown on the right, where pose $\mathbf{P_i}$ is close to $\mathbf{P_{i-1}}$. Overlapping transducer poses between multiple sweeps increase the possibility of poses re-registering on already registered viewpoints. The reconstruction results may differ between iterations if camera pose parameters are incorrectly optimized and mistakenly focus on the same scene area, causing incorrect reconstructions.

Gradient-based optimization is susceptible to identifying suboptimal poses [49], as multiple pixels from different sweeps are assigned to the same spatial location $x_j$. Misalignment occurs between sweeps where different subsets of the transducer trajectory observe the same part of the 3D scene, resulting in reconstruction distortions. As shown in figure 4, the optimization incorrectly associates subsets of the pose trajectory with the same area of the radiance field, resulting in overlapping registration. More specifically, it could provide an incorrect update with the term $\frac{\delta \mathbf{y_i}\left[\mathbf{r_i}\left(h\right)\right]}{\delta \mathbf{r_i}\left(h\right)}$ from equation (9) during backpropagation, create gradient fluctuations and push the model away from the optimal neural field. To find the optimal transducer pose $\textbf{T}^\ast$, we propose to place constraints on $\frac{\delta \mathbf{y_i}\left[\mathbf{r_i}\left(h\right)\right]}{\delta \mathbf{r_i}\left(h\right)}$ for the smoothness of $\textbf{y}$.

### 3.4. Geometry-aware US NeRFs

We now introduce GAU-NeRF, a simple yet effective NeRF-based model for freehand US reconstruction that prevents updates to suboptimal transducer poses. We propose to promote the smoothness of $\textbf{y}$ by re-weighting the Jacobian matrix $\textbf{G} = \frac{\delta \mathbf{y_i}\left[\mathbf{r_i}\left(h\right)\right]}{\delta \mathbf{r_i}\left(h\right)}$. Inspired by geometric Polyak–Ruppert averaging [34], stochastic re-weighted gradient descent [54], and the coarse-to-fine positional encoding annealing strategy [31], we re-weight $\textbf{G}$ by

\begin{equation*} w_k\textbf{G}+\left(1-w_k\right)\bar{\textbf{G}}.\end{equation*}

The weight parameter $w_k = \frac{k}{L}$ is defined as

\begin{equation*} w_k = \begin{cases} 0, &amp;\frac{k}{L} < q \\ \frac{k}{L}, &amp;\frac{k}{L} \unicode{x2A7E} q \\ \end{cases}\end{equation*}

where $k$ is the current optimizing iteration, $L$ is the total number of iterations, and $q$ is a hyperparameter proportional to the optimization progress. $\bar{\textbf{G}}$ is the average gradient between the current gradient at iteration $k$ and iteration $k-1$ for $k > 0$,

\begin{equation*} \bar{\mathbf{G_k}} = \frac{1}{2}\left(\mathbf{G_{k}}+ \mathbf{G_{k-1}}\right).\end{equation*}

Starting from the raw 3D input $(k = 0)$, we only use the weighted average gradient along the ray until the optimization process reaches $q$, then gradually reduce the portion of $\bar{\textbf{G}}$ to decrease the impact of large spikes of gradient update at the earlier training stage.

### 3.5. Implementation details

Our model is trained from scratch via stochastic gradient descent to fit an input scene. We build GAU-NeRF by utilizing the coarse-to-fine registration scheme from BARF [31] and the rendering model from Ultra-NeRF [25]. MSE is used to account for photometric loss. Inspired by separable collaborative filters [55], we first parametrize the PSF that is initialized as a standard Gaussian. We then use single value decomposition to decompose into two learnable 1D vectors $\mathbf{k_w}\in{\mathbb{R}}^{n\times W} $and $\mathbf{k_h}\in{\mathbb{R}}^{n\times H}$. $n$ denotes the kernel size and is set to 3, and $H,W$ represent the height and width of the input 2D US images, respectively.

We implement our model in PyTorch with 8 MLP layers with 256 channels per layer. It is trained with Adam optimizer [56] $(\beta\ = [0.9, 0.99], \epsilon = {10}^{-9}$, weight decay ${10}^{-5}$, and learning rate ${10}^{-3}$). We also modify ray sampling from equidistant to the stratified method to capture diverse scenes and minimize artifacts. We train all models for $L = 200,000$ iterations and randomly sampled 16 384 rays at each learning step. The controllable parameter for coarse-to-fine registration is linearly modified following BARF [31], transitioning from iteration 20 000 to 100 000. The hyperparameter $q$ is set to 0.4. With a single NVIDIA A40 GPU (NVIDIA, Santa Clara, USA), our method takes approximately two hours to train. We found that increasing the weight decay and learning rate could further accelerate training to be under 30 minutes for generating slightly lower-quality images. We also consider a realistic US reconstruction scheme called continuous Bernoulli (CB) [57]. In our previous work, we utilized a CB distribution to estimate the scatterer distribution [57].

## 4. Results

In this section, we describe a comprehensive evaluation of our method using implicit reconstructions from imperfect transducer poses. We performed experiments on three different datasets and also compared our method to other NeRF methods.

### 4.1. Data collection

We use the synthetic liver dataset [25], in-house curated *in vivo* TM phantom, and *in vivo* knee datasets [24] for training and validating our model. Synthetic liver data were utilized for evaluating both novel-view reconstruction performance and camera pose recovery, while *in vivo* datasets were employed for assessing the reconstruction performance. The synthetic liver dataset from [25] includes a total of 1400 2D US images (200 images per sweep), each with a size of 512 by 256 pixels. Since the synthetic liver dataset assumes no registration mismatch between sweeps, to simulate realistic transducer pose errors, we generated training poses by adding random noise ($U \sim (-2,2)$) to the GT poses. The average translation error is $9.8 \mathrm{mm}$ and the average rotation error is $11.57^{\circ}$.

For the TM dataset, we acquired two freehand datasets on a TM abdominal phantom (Model 057A, CIRS Inc. USA) using a linear array transducer (10L4) on a Siemens Sequoia US system (Siemens Medical Solutions, USA) while tracking the transducer location. The transducer’s pose was tracked using a 6D EM tracking system (NDI 3D Guidance, Northern Digital Inc. Canada), with the tracking sensor securely mounted to the transducer via a tracking bracket (CIVCO Medical Solutions, USA) to maintain consistent spatial alignment between the transducer and image plane. The EM tracking system was calibrated according to the manufacturer’s specifications. For calibration, we used the Velmex slides (Velmex, USA) and the ImFusion Suite Calibration Wizard (ImFusion GmbH, Germany; version 3.8.1). The Velmex slides documenting the movement of the transducer, scanning on a multi-purpose phantom (Model 040GSE, CIRS Inc. USA). Each dataset contains four sweeps captured at varying angles relative to the phantom surface, containing 621 and 865 US images, respectively, each sized at 256 $\times$ 512 pixels. This is to ensure that the underlying scenes are captured from different viewing angles. We also used two *in vivo* knee datasets with a total of 1572 images at 596 by 520 pixels, with the detailed acquisition protocol presented in [24].

### 4.2. Experiment protocol and evaluation metrics

For training, we utilized a training/testing dataset split. More specifically, we used 800 images for training and 600 for testing from the synthetic liver data. For TM dataset, 1000 images were used for training and 486 for testing. We also used 1172 images and 400 images for training and testing, respectively for the *in vivo* knee dataset. For pose estimation, we computed the rotation error as

\begin{equation*} \arccos\left(\ \frac{tr\left(\textbf{R}^\top \mathbf{R_{gt}}\right)-1\ }{2}\right).\end{equation*}

$\mathbf{R_{gt}}$ is the GT rotation while $\textbf{R}$ is the prediction, and the operation $tr\left(\cdot\right)$ computes the trace of a matrix. The translation error was computed using the l2-norm between the estimated $\textbf{t}$ and GT $\mathbf{t_{gt}}$ as:

\begin{equation*} \| \ \textbf{t}-\ \mathbf{t_{gt}} \|_2.\end{equation*}

Rotation and translation errors were only reported on the synthetic liver dataset, where ground-truth transducer poses are known.

We employed three evaluation metrics to evaluate our model against other baselines, including: the PSNR, the SSIM, and the LPIPS [58]. PSNR quantifies the ratio between the maximum possible signal power and the power of noise, measured in decibels (dB), with higher values indicating better reconstruction. SSIM measures the perceived quality of images by comparing structural information, rather than raw pixel differences. SSIM scores range from 0 to 1, where 1 indicates perfect similarity. LPIPS focuses on the global structure and noise levels of the images, effectively capturing perceptual differences and artifacts, and has been shown to correlate strongly with human visual perception when compared to PSNR and SSIM [59]. Following the standard evaluation protocol for NeRF and Ultra-NeRF [25, 60], we assess the quality of 3D reconstruction through novel view synthesis. This is done by comparing the rendered 2D US images against GT using transducer poses from a hold-out test set^5^5Freehand scanning protocol involves probe trajectories in one direction [40]. Consequently, we do not have GT in the other direction (like orthogonal) for comparison. Instead, we evaluate the 3D reconstruction quality by synthesizing 2D images from unseen transducer locations during training..

For baseline measurement, we compare our results with the NeRF baselines including NeRF [20], and optimization-based pose recovery methods L2G-NeRF [49], BARF [31], and Ultra-NeRF [25]. We also evaluated our model against Deblur-NeRF [61] to determine if the reconstruction result could be improved by learning a deblurring kernel.

### 4.3. Evaluation on simulated datasets

**Figure 5.**
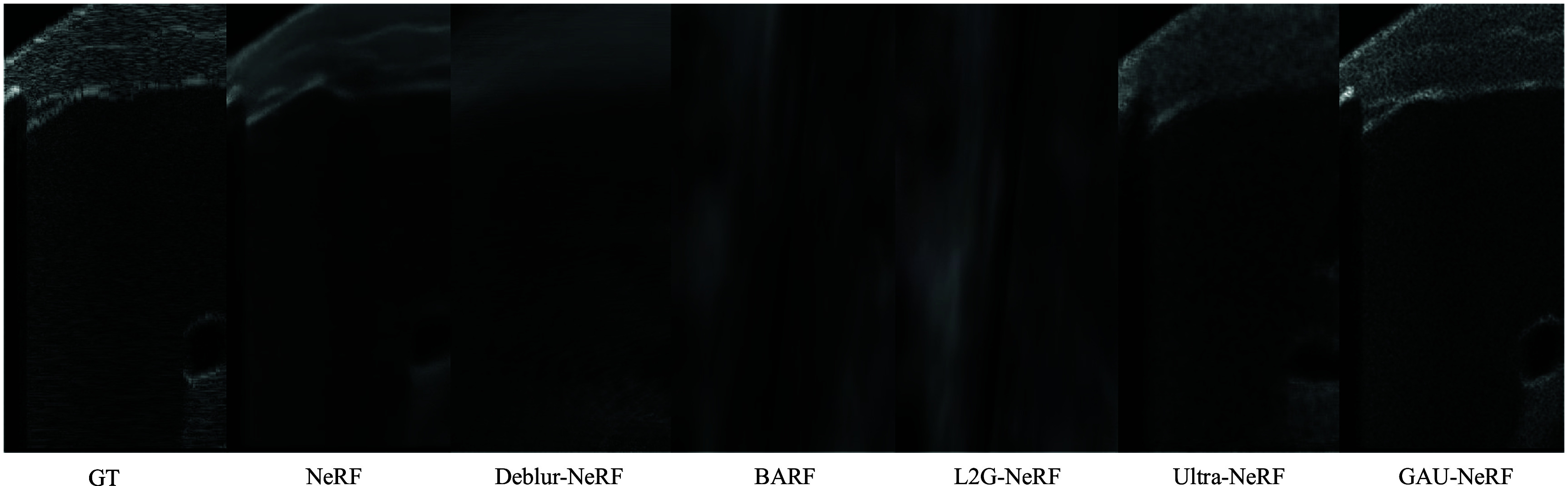
Comparison of reconstructions against ground-truth ultrasound data on simulated liver data.

Table 1 shows the comparison of reconstruction quality on the simulated liver dataset, evaluated by rendering 2D US images and computing PSNR, SSIM, and LPIPS metrics against GT B-mode images. Higher PSNR and SSIM indicate better reconstruction, while lower LPIPS indicates better perceptual similarity. Note that our model improves the reconstruction quality in terms of PSNR, SSIM, and LPIPS. Although Ultra-NeRF attains competitive PSNR, SSIM, and LPIPS scores, it does not match the overall performance of GAU-NeRF or NeRF. This suggests that the single-state training approach used by Ultra-NeRF could have a negative impact on scene reconstruction, since it does not progressively refine a low-resolution representation before moving to a high-resolution one. Figure 5 shows the visualization results for the reconstruction. Note that BARF and L2G-NeRF fail to learn the neural fields. GAU-NeRF scored the lowest LPIPS on the simulated dataset, further suggesting that our model produces higher perceptual quality US images. Even though L2G-NeRF has a slightly lower rotation error, it does not find better solutions based on reconstruction quality and visualization results in figure 5. When first-order gradients are used to update the poses jointly, a range of smoothed yet incorrect neural fields can still emerge, even when highly implausible structures are disregarded. These fields may perfectly fit the GT transducer poses but exhibit poor reconstruction quality [29, 30, 33]. Ultra-NeRF retrains some of the structures but introduces artifacts due to inaccurate transducer poses. BARF and L2G-NeRF fail to reconstruct the 2D image. In contrast, our GAU-NeRF preserves textural details in alignment with GT. These qualitative results are consistent with the quantitative results shown in table 1.

**Table 1.** Quantitative results with the simulated liver datasets. N/A: Rotation and translation results are not available since those methods do not recover spatial position information. Translation errors are scaled by 100.

	Rotation (degree)	Translation (mm)	PSNR (dB)	SSIM	LPIPS
NeRF	N/A	N/A	$27.26 \pm 1.39$	$0.55 \pm 0.02$	$0.65 \pm 0.03$
L2G-NeRF	**2.17** $\pm$ **0.01**	$13.98 \pm 1.57$	$15.05 \pm 0.37$	$0.51\pm 0.01$	$0.75 \pm 0.01$
Deblur-NeRF	N/A	N/A	$22.23 \pm 1.29$	$0.34\pm 0.03$	$0.78 \pm 0.03$
BARF	$2.19 \pm 0.02$	$141.37 \pm 0.44$	$19.39 \pm 0.71$	$0.46\pm 0.02$	$0.80 \pm 0.04$
Ultra-NeRF	N/A	N/A	$25.25 \pm 0.89$	$0.35 \pm 0.02$	$0.51 \pm 0.03$
GAU-NeRF	$2.27 \pm 0.53$	**7.63**$ \pm$ **0.16**	**28.58** $\pm$ **0.41**	**0.61** $\pm$ **0.03**	**0.23** $\pm$ **0.02**

### 4.4. Evaluation on *in vivo* datasets

**Figure 6.**
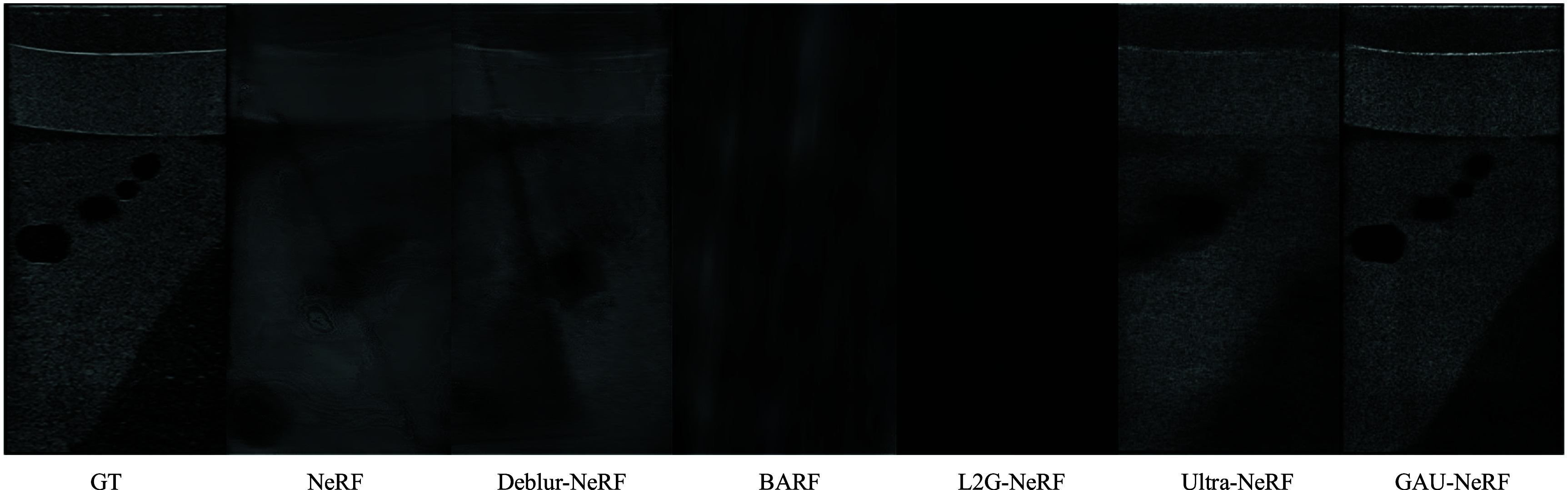
Reconstruction results from data acquired on a TM experimental phantom.

**Figure 7.**
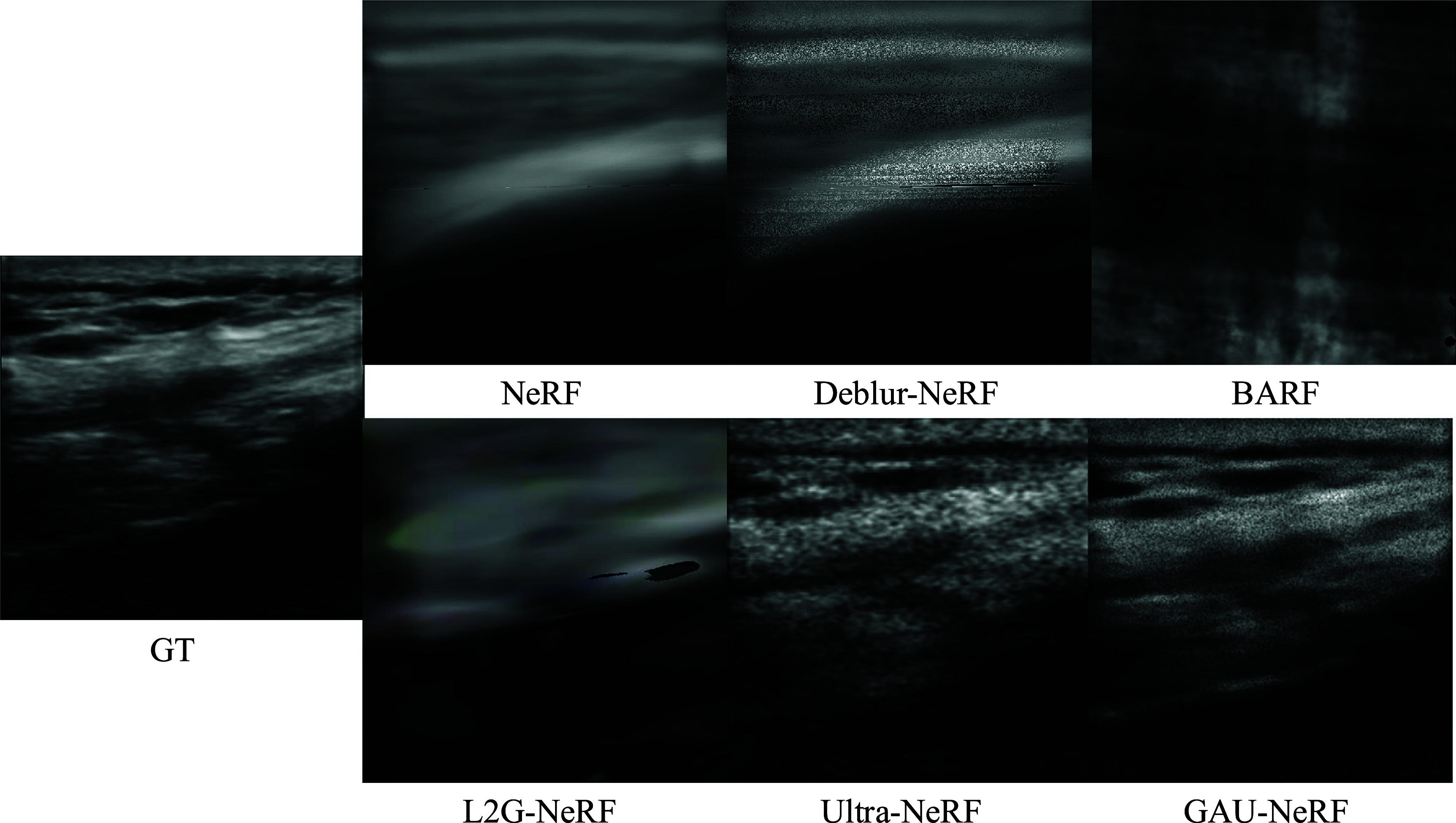
Reconstruction results from the *in vivo* knee dataset.

Table 2 summarizes the quantitative results on the *in vivo* knee datasets, along with the experimental TM phantom datasets. GAU-NeRF achieves the best results across all three metrics. NeRF and Deblur-NeRF are unable to learn a high-quality neural field without accurate transducer poses. Results from figures 6 and 7 further highlight the ability of GAU-NeRF to recover transducer pose information and reconstruct neural fields from scratch with the real data, where BARF and L2G-NeRF are unable to recover the underlying scene from inaccurate poses. We note that GAU-NeRF does not preserve all anatomical details, especially for complicated structures. One explanation can be attributed to the freehand sweep mechanism itself, since sonographers/physicians could miss regions that are hard to access with transducers during acquisition.

**Table 2.** Quantitative results with the TM phantom and the knee datasets.

TM phantom			

	PSNR (dB)	SSIM	LPIPS
NeRF	$21.45 \pm 0.05$	$0.41 \pm 0.01$	$0.78 \pm 0.03$
L2G-NeRF	$12.59 \pm 0.40$	$0.44\pm 0.04$	$0.77 \pm 0.01$
Deblur-NeRF	$21.04 \pm 1.11$	$0.45\pm 0.05$	$0.78 \pm 0.01$
BARF	$13.98 \pm 0.02$	$0.45\pm 0.04$	$0.78 \pm 0.01$
Ultra-NeRF	$25.02 \pm 1.36$	$0.35 \pm 0.03$	$0.51 \pm 0.02$
GAU-NeRF	**29.39** $\pm$ **0.51**	**0.49** $\pm$ **0.01**	**0.18** $\pm$ **0.02**

Knee			

	PSNR (dB)	SSIM	LPIPS

NeRF	$15.29 \pm 0.71$	$0.61 \pm 0.03$	$0.79 \pm 0.02$
L2G-NeRF	$16.06 \pm 1.56$	$0.57 \pm 0.03$	$0.72 \pm 0.02$
Deblur-NeRF	$14.15 \pm 0.83$	$0.32 \pm 0.02$	$0.77 \pm 0.02$
BARF	$12.37 \pm 0.87$	$0.44\pm 0.04$	$0.77 \pm 0.02$
Ultra-NeRF	$24.75 \pm 1.32$	$0.58 \pm 0.02$	$0.34 \pm 0.02$
GAU-NeRF	**28.65** $\pm$ **0.95**	**0.65** $\pm$ **0.01**	**0.29** $\pm$**0.02**

### 4.5. Ablation studies

Finally, we perform ablation studies to understand why our proposed GAU-NeRF provides significant improvements in terms of reconstruction. The first analysis focused on the effectiveness of the CB distribution on the TM phantom dataset and the impact of a vanilla implementation of BARF combined with the Ultra-NeRF without reweighting. The naive implementation is denoted as Ultra-NeRF + BARF, and the method utilized for estimating scatterer density from Ultra-NeRF in GAU-NeRF used as the baseline for comparison, denoted as GAU-NeRF without CB.

**Figure 8.**
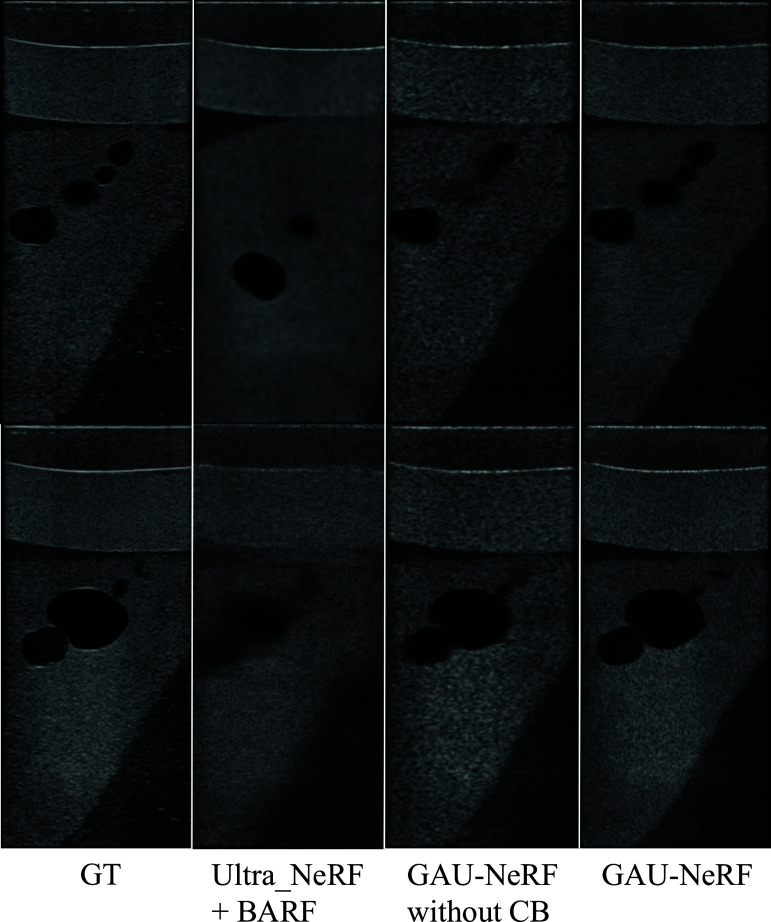
Reconstruction results from a TM experimental phantom. Using CB distribution, GAU-NeRF can recover underlying scenes that are closer to the ground truth.

Quantitatively, our model achieves better reconstruction results, as shown in table 3, demonstrating improved scatterer localization. Moreover, the result shows that although BARF improves registration results, the optimization still falls into a suboptimal local minimum. Qualitative visualization in figure 8 suggests that GAU-NeRF achieves better reconstruction of the underlying B-mode image utilizing a CB distribution to estimate the scatterer distribution. These results suggest that our design is effective, and CB can further boost the reconstruction quality.

**Table 3.** Ablation study results with the phantom data.

TM phantom			
	PSNR (dB)	SSIM	LPIPS
Ultra-NeRF	$25.02 \pm 1.15$	$0.49 \pm 0.02$	$0.23 \pm 0.02$
Ultra-NeRF + BARF	$24.31 \pm 1.15$	$0.34 \pm 0.02$	$0.27 \pm 0.02$
GAU-NeRF without CB	$27.64 \pm 0.92$	$0.51 \pm 0.03$	$0.21 \pm 0.02$
GAU-NeRF	**28.65** $\pm$ **0.85**	**0.60** $\pm$ **0.01**	**0.18** $\pm$**0.01**

We also tested the impact of the hyperparameter $q\in\left[0.4,0.5,0.6\right]$ on the model’s performance. Table 4 shows the ablation results of GAU-NeRF on the TM dataset. Reweighting the gradient at an earlier stage may improve LPIPS, but the PSNR score worsens on this dataset. However, it could help guide the model to the correct registration without large gradient fluctuations.

**Table 4.** Ablation study results on the hypermeter **q**.

TM phantom			
q	PSNR (dB)	SSIM	LPIPS
0.4	$28.47 \pm 0.37$	** $0.49 \pm 0.01$ **	** $0.16 \pm 0.02$ **
0.5	**28.61** $\pm$ **0.52**	**0.49** $\pm$ **0.01**	**0.17** $\pm$ **0.07**
0.6	$28.53 \pm 0.51$	**0.49** $\pm$ **0.01**	$0.23 \pm 0.02$

### 4.6. Time complexity comparison

We further report training and inference time of our GAU-NeRF. All processing time comparison experiments were performed on the same hardware using the phantom dataset. As shown in table 5, our model takes approximately 2.5 h to train and 50 s for testing on the phantom dataset. It achieves comparable time compression to other baseline models. Time analysis indicates that calculating the gradient via reweighting incurs negligible overhead for our model.

**Table 5.** Average training time (hh:mm) and inference time (mm:ss) on the TM phantom data.

Training					
NeRF	BARF	L2G-NeRF	Deblur-NeRF	Ultra-NeRF	GAU-NeRF

01:30	02:21	01:32	3:35	01:43	02:23

Inference					

0:39	0:51	0:49	1:01	00:43	0:50

## 5. Discussion and conclusion

In this paper, we introduce GAU-NeRF, a NeRF-based method for freehand 3D US reconstruction. It builds upon NeRF, and addresses a critical challenge in freehand US, namely suboptimal reconstruction result due to the inaccuracy of transducer pose information. We also identify key challenges in the joint optimization of scene representation and pose estimation, which can degrade reconstruction quality. To address this challenge, we propose an optimization scheme that incorporates smoothening regularization when updating transducer poses during training. Our method leads to more accurate transducer poses, and thus significantly improved reconstruction quality. GAU-NeRF achieves strong empirical results on both simulated and *in vivo* US datasets, outperforming baseline methods by a substantial margin. These findings position GAU-NeRF as a promising method toward practical, clinic-ready solutions for freehand 3D US reconstruction.

While GAU-NeRF shows strong results, it inherits certain limitations from the original BARF formulation, including slow optimization and rendering, sensitivity to the quality of dense 3D sampling, and reliance on heuristic geometry-aware constraints, which can introduce inefficiencies. Also, structural details may be missing from *in vivo* datasets due to operator error, if the underlying structure is not fully captured during US data acquisition. However, given the structural similarities between our model and NeRF, recent advancements including the use of foundation models to recover the missing anatomical structures in NeRF [62], can be readily adapted to enhance our model’s performance. One possible future direction is to incrementally reconstruct the local geometry while refining poses for unposed image sequences. For example, an adaptive tetrahedral interpolation method can be used to provide local geometry cues for optimization [63]. Moreover, neural rendering approaches for monocular endoscopy reconstruction, such as [64], which supervise geometry with a learned prior, can accelerate optimization at the same time reduce drift through SLAM-style tracking.

Our GAU-NeRF approach has the potential to substantially extend 3D US functionality using existing US 1D array systems without major overhead, e.g. acquiring a dedicated US system or matrix array transducers. It thus opens exciting opportunities for 3D reconstructions in point-of-care settings with the use of current clinical systems for 3D imaging.

## Figures and Tables

**Figure 9.**
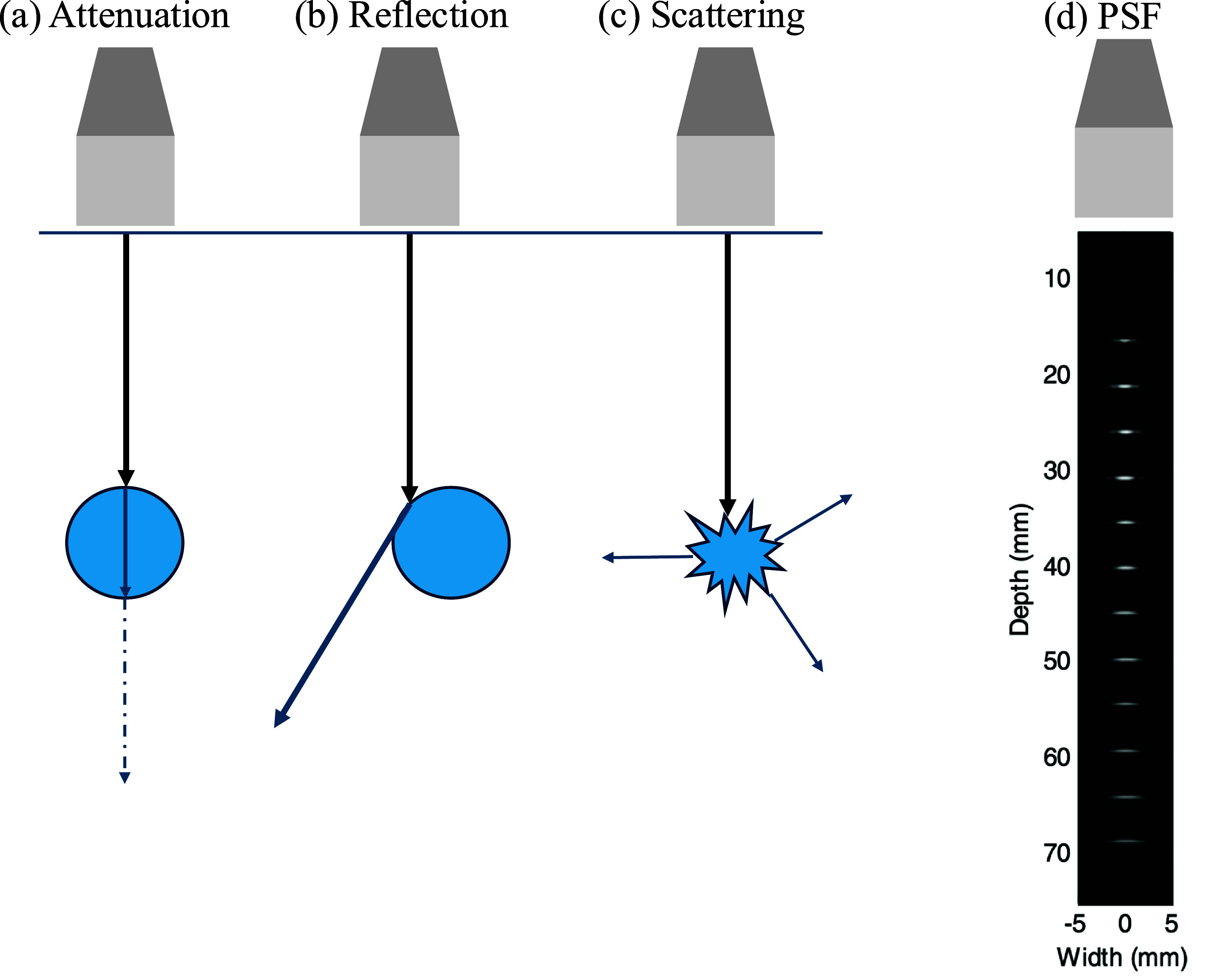
US images are formed by acoustic wave interactions with tissue, involving:(a) attenuation, (b) reflection, (c) scattering, and (d) PSF, the system’s spatial resolution. $PSF(\textbf{r})$ is simulated with Field II [65] at various depths for a 4 MHz center frequency, using the model with 32 by 32 elements. For ray propagation, US rays emanated from the transducer origin $o$ with initial intensity, sampling query points at depths $h$ where physics parameters are evaluated.

## Data Availability

The liver dataset used in the paper was obtained from [25]. The knee dataset was from [24] where the authors stated that the data acquisition followed institutional REB (Research Ethics Board) guidelines. The data that support the findings of this study will be openly available following an embargo at the following URL/DOI: https://github.com/Alphafrey946/GAU_NERF. [68] Data will be available from 31 December 2026.

## Appendix

### A.1. US wave interaction with tissues

The formation of US images is governed by the interaction between acoustic waves and biological tissue, as illustrated in figure 9. When US waves propagate through the tissue, they undergo three primary physical phenomena that determine image quality, including attenuation, reflection, and scattering. We leverage a ray-tracing-based technique [50] to enable NeRF for the US rendering task. Figure 9(a) describes the gradual reduction in terms of the US wave intensity, propagating through different medium. This depth-dependent intensity loss is parameterized by the attenuation coefficient $\boldsymbol{\alpha}$. Figure 9(b) demonstrates the reflection, at tissue boundaries where abrupt acoustic impedance changes occur, such as organ interfaces or vessel walls. The reflect coefficients (range from 0 to 1) are denoted as $\boldsymbol{\beta}$. Figure 9(c) illustrates the scattering that arises when the US wave hits microscopic tissue inhomogeneities smaller than the acoustic wavelength, producing the speckle pattern in US images [66]. Unlike directional reflection, scattered wave propagates omnidirectionally, characterized by the scattering density $\mathbf{p}_{\mathbf{b}}$ and the scattering intensity $\mathbf{p}_{\mathbf{s}}$. $PSF(\textbf{r})$ parameterized the impulse response of a US system response to a point target at depth $\textbf{r}$. Figure 9(d) shows a typical US transducer with better lateral resolution near the focal zone and degraded axial resolution at depths, causing blur [67]. In our GAU-NeRF framework, these physics parameters $\left[\boldsymbol{\alpha},\boldsymbol{\beta},\mathbf{p}_{\mathbf{b}},\mathbf{p}_{\mathbf{s}},\boldsymbol{\phi}\right]$ and $PSF$ are explicitly modeled as learnable parameters, enabling physically accurate rendering that accounts for US specific imaging characteristics absent in the vanilla NeRF formulation, ensuring reconstruction quality.
